# F255. FACTORS RESPONSIBLE FOR DELAY IN TREATMENT SEEKING IN PATIENTS WITH PSYCHOSIS- A QUALITATIVE STUDY FROM CENTRAL INDIA

**DOI:** 10.1093/schbul/sby017.786

**Published:** 2018-04-01

**Authors:** Mamidipalli Spoorthy

**Affiliations:** All India Institute of Medical Sciences, Raipur

## Abstract

**Background:**

Delay in treatment seeking in psychoses is not only influenced by stigma, societal attitudes, unawareness, under-diagnosis but also is coloured by the socio-cultural background of the patient. Finding out these reasons for delay in treatment can help both the patient and the family members by reducing the morbidity & burden associated with untreated psychosis.

**Methods:**

This is a hospital based cross-sectional study, conducted Raipur. Included are purposive sample of 25 family members & patients with a diagnosis of schizophrenia, schizoaffective disorder, or psychotic disorder-not otherwise specified - using the Mini International Neuropsychiatric Interview-Plus version, aged 18–60 years, who are able to understand and speak Hindi, and in regular contact with the patient. DUP is defined as the number of months from the onset of positive psychotic symptoms until start of proper treatment. Semi-structured interview was conducted by using open ended questions to assess the factors responsible for treatment delay and verbatims were recorded.

Qualitative analysis

We used content analysis for the purpose of this study. Each investigator generated separate categories and themes after reading the transcripts word by word. Theme generation was continued till theoretical saturation emerged and. Categories and themes identified by both the investigators in common were used in the results as it would increase their validity.

**Results:**

1. Socio-demographic profile

64% of patients were diagnosed with Schizophrenia and the rest were diagnosed with Psychosis NOS. Mean total duration of untreated psychosis was 15 months. Relation of family members with the patient was like parents (48%), spouse (24%), siblings (12%), children (8%), uncle/aunt (4%), grand-parents (4%).

2. Results of qualitative analysis

Based upon the content analysis technique used, we have generated certain categories of factors responsible for treatment delay and generated themes in each category.

A. Illness related factors

a. Unawareness of illness

b. Explanatory models of illness

i. Supernatural causation of illness

ii. Biological causation of illness

c. Stigma associated with illness

B. Patient related factors

a. Underlying pre-morbid personality

b. Symptoms at the onset

c. Onset along with life events

d. Poor insight/uncooperative patient

e. Impaired functioning

C. Family related factors

a. Shared societal beliefs

b. Cultural constraints

c. Lack of support from significant others or poor social support

D. Treatment related factors

a. Poor knowledge of general physicians about psychiatric disorders and poor referral

b. Misconceptions about the effects of medication

E. Others

a. Financial constraints

**Discussion:**

The most common cause of delay is unawareness about the illness apart from the supernatural causation. To our knowledge this is the first study where we found that if the patient’s personality presents in an exaggerated way, or patient’s psychopathology is in line with the socio-cultural background, it might lead to delay. Though the findings about patient’s poor insight, uncooperativeness, negative symptoms, absence of violence, financial burden, stigma, lack of social support was proved by many studies, preserved functioning is our novel finding.

Though these themes seem to be separate, they are interdependent and interact in a complex way leading to the delay in treatment seeking. Interventions focused at each and every step need to be devised in further studies in order to overcome these barriers.

